# Editorial on the Special Issue “Advances in Cellulose-Based Hydrogels”

**DOI:** 10.3390/gels8120790

**Published:** 2022-12-02

**Authors:** Lorenzo Bonetti, Christian Demitri, Laura Riva

**Affiliations:** 1 Department of Chemistry, Materials and Chemical Engineering “G. Natta”, Politecnico di Milano, Via Luigi Mancinelli 7, 20131 Milan, Italy; 2 Department of Engineering for Innovation, Campus University Ecotekne, Università del Salento, Via per Monteroni, 73100 Lecce, Italy

Cellulose is one of the most ubiquitous and naturally abundant biopolymers found on Earth and is primarily obtained from plants and other biomass sources. It is considered to be a nearly limitless raw material supply that is able to meet the rising demand for ecologically friendly and biocompatible products, with a huge annual production worldwide (~1.5 × 10^12^ tons) and a capacity for extraction even from waste sources. Cellulose-based hydrogels are particularly appealing for both the academic and industrial fields, since they often combine hydrophilicity, biodegradability, non-toxicity, and biocompatibility with a low cost and wide availability. Biomedical engineering, advances in smart systems (such as sensors, actuators, and soft robotics), stimuli-responsive systems (such as pH- or thermo-responsive hydrogels), the agricultural industry (such as soil conditioning, nutrient carriers, and water reservoirs), and water purification are just some of the potential areas of application.

This field of research has continuously and exponentially grown over the past decade, as demonstrated by the rising number of annual publications on cellulose-based hydrogels, which amount to only 8 in the 2000s versus 497 in 2022 (Scopus database). This book includes 15 papers and reviews covering a wide variety of cellulose-based hydrogel applications, ranging from drug delivery to heterogeneous catalysis. Together, these papers provide a valuable overview of the fields engaged in research on cellulose-based hydrogels, offering a perspective on the challenges and opportunities for future development in this area.

In recent years, cellulose-based hydrogels have attracted significant attention in the biomedical field owing to both their outstanding intrinsic properties (e.g., biocompatibility and degradability) and the possibility of obtaining designable functions through different preparation methods and structure designs. In this regard, the review conducted by Zou et al. [1] covers the recent advances in research on cellulose-based hydrogels, introducing their applications and future developments in the field. In the same vein, the paper of Filip et al. describes self-assembled gels based on natural deep eutectic solvents and hydroxypropyl cellulose, with strong antibacterial and antifungal activities [2]. These systems were tested in vitro in the presence of *S. aureus*, *E. coli*, *P. aeruginosa*, and *C. albicans*, confirming their antimicrobial activity and showing a good biocompatibility with primary cells (human gingival fibroblasts). In their work, Blažic et al. synthesized and fully characterized cellulose-g-poly (2-(dimethylamino)ethylmethacrylate) hydrogels [3]. The obtained hydrogels showed a weak antibacterial activity in vitro against *E. coli*, *P. aeruginosa*, and *B. subtilis*. However, cyclic voltammetry (CV) and electrochemical impedance spectroscopy (EIS) were also performed, demonstrating that these systems, despite their weak antibacterial performance, have good characteristics as supercapacitors. A novel hydrogel based on polyvinyl alcohol (PVA), carboxymethyl cellulose (CMC), and polyethylene glycol (PEG) was designed by Yang et al. for the growth of zeolitic imidazolate framework-L (ZIF-L) in situ [4]. The release of Zn^2+^ and imidazolyl groups elicited a synergistic antibacterial activity against *S. aureus* in vitro. At the same time, the obtained hydrogel promoted cell proliferation at an early stage, enhancing its coagulation efficiency. A rat liver injury model was further employed to confirm the rapid hemostasis capacity of the developed hydrogel.

Cellulose-based hydrogels have successfully been employed as delivery systems to improve the controlled release of particles and (bio)molecules, even as a function of physiological and/or pathological stimuli (e.g., temperature and pH variations). In this regard, Bonetti et al. [5] designed a novel methylcellulose (MC)-based hydrogel for the controlled release of silver nanoparticles (AgNPs) in infected chronic wounds, characterized by a local pH increase. The obtained MC hydrogels showed swelling and degradation behaviors that were dependent on both the pH and temperature, and a noteworthy pH-triggered release of AgNPs (release ~10 times higher at pH = 12 than pH = 4). In their work, Liu et al. [6] fabricated innovative electrospun core–shell nanohybrids composed of hydroxypropyl methylcellulose (HPMC) and acetaminophen (AAP) in the core sections and composites of polyvinylpyrrolidone (PVP) and sucralose in the shell sections. Interestingly, HMPC gelation ensured a faster release of AAP, which could be beneficial for potential orodispersible drug delivery applications. Shaik et al. [7] reported an oleogel-based bigel derived from a guar gum hydrogel and a sesame oil/candelilla wax, which they further tailored using date palm-derived cellulose nanocrystals (dp-CNC). The addition of dp-CNC as a novel reinforcing agent allowed these bigels to act as carriers of moxifloxacin hydrochloride (MH).

The biomedical applications of cellulose-based hydrogels are not limited to the above-mentioned studies but can also be extended to the field of conductive hydrogels. In this regard, Kasaw Gebeyehu et al. [8] present a review of the current state of the art of conductive hydrogel manufacturing based on cellulosic materials used for tissue engineering, in addition to reporting the current scenario and the possible future developments in the field. In this regard, Jin et al. [9] report on conductive hydrogel inks consisting of CMC, tannic acid, and metal ions (HAuCL_4_) developed for on-skin direct printing. These hydrogels showed self-healing properties and reversible conductivity under cyclic strain, and they were successfully on-skin printed, achieving the continuous electrical flow of the electronic circuit under the conditions of skin deformation. In their work, Park et al. [10] present a soft stretchable conductive hydrogel composite consisting of alginate (Alg), CMC, polyacrylamide (PAM), and silver flakes (AgF). The obtained hydrogel fully supported the operation of a light-emitting diode under the conditions of mechanical deformation and successfully enabled the measurement of electromyogram signals.

Another important area in which cellulose-based hydrogels have found application is photo-catalysis, with a focus on water remediation. Photocatalytic systems hold great promise as innovative alternatives to common environmental decontamination systems, and the review conducted by Yang et al. [11] extensively describes the recent advances in cellulose-based hydrogel photo-catalysts, first discussing the properties and preparation methods and then classifying these systems according to the type of catalyst and the research progress in different fields. In the context of sustainable technological development, the work of Riva et al. [12] reports on hydrogel-derived nano-sponges that are capable of capturing heavy metal ions from aqueous solutions. Based on their observations of this interesting property, the authors describe the application of these materials in the field of heterogeneous catalysis, successfully catalyzing the formation of aromatic acetals using nanosponges loaded with Cu^2+^ and Zn^2+^ metals and achieving extremely high yields and a very good selectivity for the desired products.

Cellulose-based hydrogels can also find application in other industrial fields, such as food packaging and the agricultural sectors. In this regard, the work conducted by Fujita et al. [13] investigates the production of a biodegradable alternative to polyacrylic-acid-based superabsorbent spheres, producing spherical hydrogel particles from CMC that could be useful in industrial and agricultural applications. Meanwhile, Jo et al. [14] report on the preparation of cellulose pulp (CP), polyurethane (PU), and curcumin-based composite films using a cost-effective method based on the use of N-methylmorpholine N-oxide (NMMO) as a solvent. The obtained composite films presented good antioxidant activities and the absence of cytotoxic effects on a HaCat cell line in vitro, thus lending themselves well to packaging and biomedical applications.

Lastly, Zitzmann et al. [15] propose a novel analytical tool for visualizing the cellulose content in defibrillated cellulose derived from microalgae using Carbotrace 480. Exploiting the distinctive fluorescent properties of the optotracer, this study provides a means through which to correlate the cellulose content in the samples with the successful hydrogel formation.
